# Cloning and expression of an endo-1,4-β-xylanase from the coffee berry borer, *Hypothenemus hampei*

**DOI:** 10.1186/1756-0500-5-23

**Published:** 2012-01-10

**Authors:** Beatriz Padilla-Hurtado, Claudia Flórez-Ramos, Carolina Aguilera-Gálvez, Jefferson Medina-Olaya, Andrés Ramírez-Sanjuan, José Rubio-Gómez, Ricardo Acuña-Zornosa

**Affiliations:** 1 Disciplina de Mejoramiento Genético, Centro Nacional de Investigaciones de Café (CENICAFE), Planalto, Km 4 vía antigua Chinchiná-Manizales, Chinchiná, Colombia; 2 Disciplina de Fisiología Vegetal; Centro Nacional de Investigaciones de Café (CENICAFE), Planalto, Km 4 vía antigua Chinchiná-Manizales, Chinchiná, Colombia

## Abstract

**Background:**

The coffee berry borer, *Hypothenemus hampei*, reproduces and feeds exclusively on the mature endosperm of the coffee seed, which has a cell wall composed mainly of a heterogeneous mixture of hemicellulose polysaccharides, including arabinoxylans. Xylanases are digestive enzymes responsible for the degradation of xylan based polymers, hydrolyzing them into smaller molecules that are easier to assimilate by insects. We report the cloning, expression and enzymatic characterization of a xylanase gene that was identified in the digestive tract of the coffee berry borer.

**Methods:**

The complete DNA sequence encoding a *H. hampei *xylanase (*HhXyl*) was obtained using a genome walking technique in a cDNA library derived from the borer digestive tract. The *XIP-I *gene was amplified from wheat (*Triticum aestivum *variety Soisson). A *Pichia pastoris *expression system was used to express the recombinant form of these enzymes. The xylanase activity and XIP-I inhibitory activity was quantified by the 3,5-dinitrosalicylic (DNS). The biological effects of XIP-I on borer individuals were evaluated by providing an artificial diet enriched with the recombinant XIP-I protein to the insects.

**Results:**

The borer xylanase sequence contains a 951 bp open reading frame that is predicted to encode a 317-amino acid protein, with an estimated molecular weight of 34.92 kDa and a pI of 4.84. Bioinformatic analysis revealed that *HhXyl *exhibits high sequence homology with endo-β-D-xylanases of *Streptomyces bingchenggensis *from glycosyl hydrolase 10 (GH10). The recombinant xylanase showed maximal activity at pH 5.5 and 37°C. XIP-I expressed as a recombinant protein inhibited HhXyl activity *in vitro *and caused individual *H. hampei *mortality in bioassays when included as a supplement in artificial diets.

**Conclusion:**

A xylanase from the digestive tract of the coffee berry borer was identified and functionally characterized. A xylanase inhibitor protein, XIP-I, from wheat was shown to be a potent inhibitor of this xylanase, suggesting that its deployment has potential as a strategy to control coffee berry borer colonization of coffee plants.

## Background

The coffee berry borer, *Hypothenemus hampei *(Ferrari) (Coleoptera: Curculionidae), is the most important pest of coffee crops worldwide. It causes annual economic losses close to $500 million due to loss of seed weight and premature fall of the bean [1]. In Colombia, it is estimated that borer infestation affects 800,000 acres, affecting the assets of more than half a million coffee growers [2].

The insect feeds exclusively on the fruit and reproduces inside the coffee seed. Carbohydrates, in the form of polysaccharides and free sugars, comprise 50% of the dry weight of the mature seed of the coffee bean. The polysaccharides include galactomannans (25%), arabinogalactans (17%) and cellulose (8%) [3]. However, other polysaccharides are likely present, including xyloglucan and arabinoxylan, and indeed, acid hydrolysis of seed carbohydrates releases small amounts of xylose [4].

In order to degrade these polysaccharides, the coffee berry borer would need to secrete a range of glycosyl hydrolases (GHs) into its digestive system. Here we report the first characterization of an endo-β-1,4-xylanase (HhXyl) from the digestive tract of the coffee berry borer. Endo-β-1,4-xylanases (EC 3.2.1.8) catalyze the hydrolysis of the β-1,4-xylosidic bonds in the backbone of xylan based polymers [5]. Most of these enzymes are classified in GH families 10 and 11 based on similarities in the amino acid sequences, catalytic domains, protein folds and overall architecture [6]. Recently, the study of xylanolytic enzymes has been an area of great interest due to their numerous industrial applications [7,8] and their roles as pathogenicity factors in plant pathogenic microbes [9].

The complete *HhXyl *transcript sequence was determined from a digestive tract cDNA library, and its homology with endo-β-1,4-xylanase genes from bacteria of the genus *Streptomyces *was established. Recombinant HhXyl protein was expressed in a *Pichia pastoris *system to analyze its enzymatic activity. In addition a xylanase inhibitor protein (XIP-I) from wheat (*Triticum aestivum *variety Soisson) that inhibits endoxylanases, was cloned and similarly expressed [10]. XIP-I inhibits the microbial GH10 and GH11 families of xylanases in a competitive manner [11] as well as two α-amylases from barley [12]. The inhibitory activity of recombinant XIP-I against the borer xylanase was evaluated in biochemical and biological assays.

Given their functions as digestive enzymes, the identification and biochemical characterization of glycolytic enzymes in *H. hampei *represents an important step in designing strategies to improve resistance against the insect. Specific protein inhibitors of these enzymes are sought for use in biotechnological strategies and to generate coffee plants that are resistant to this pest.

## Results and Discussion

### Isolation and cloning of the xylanase gene from the coffee berry borer

**Figure 1:**
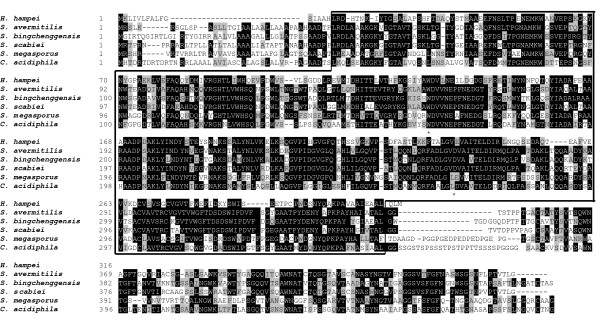
**Alignment of the deduced amino acid sequence of HhXyl of *Hypothenemus hampei *with those of other GH10 xylanases**. The sequences are as follows: β-1,4-xylanase of *Hypothenemus hampei *[GenBank:HM991729], beta-1,4-xylanase of *Streptomyces avermitilis *[GenBank:NP_826161], β-1,4-xylanase of *S. bingchenggensis *[GenBank:ADI10429.1], β-1,4-xylanase of *S. scabiei *[GenBank:YP_003489277.1], β-1,4-xylanase of *S. megasporus *[GenBank:ADE37527.1] β-1,4-xylanase of *Catenulispora acidiphila *[GenBank:YP_003115119.1] The identical and similar amino acid residues are shown in black and grey boxes, respectively. The conserved catalytic glutamate residues are indicated with asterisks. The conserved domain of the GH10 family is framed.

Of 3,070 sequenced clones in a cDNA library created from the digestive tracts of coffee berry borers, 1,252 unique sequences were obtained. Based on a BLASTX analysis, 124 Expressed Sequence Taq (ESTs) had high homology (E > 10^-4^) with corresponding sequences of proteins with known catalytic activity. Among the ESTs with putative functional annotations, 5 ESTs were found to be related to digestive proteins, one of which was classified as a putative endo-1,4-β-xylanase. Based on this EST sequence, a corresponding open reading frame of 951 bp was identified using a genome walking strategy and designated *HhXyl *[GenBank: HM991729]. This sequence is predicted to encode a 317-amino acid prot ein with an estimated molecular weight of 34.92 kDa and a pI of 4.84. Sequence analysis using the SignalP 3.0 program [13] indicated that HhXyl is a secreted protein with a peptidase recognition site between the amino acids His16 and Leu17. After removal of the signal peptide, the mature HhXyl is predicted to be a 300-amino acid protein with a molecular weight of 33.5 kDa and a pI of 4.63. BLAST analysis of the protein showed that HhXyl contains a putative domain between Leu17 and Leu313 (pfam00331) that is conserved in GH10 proteins, which typically have a molecular weight of ≥ 30 kDa and low pI [14]. In terms of substrate specificity, GH10 endoxylanases are generally less selective than those from GH11, but GH10 enzymes can hydrolyze polysaccharides that are more complex [15]. The alignment of the deduced amino acid sequence of HhXyl is shown in Figure 1, together with other GH10 endoxylanases. The functional domain of this family is highly conserved among xylanases of different species, as are the locations of residues Glu138 (acid/base catalyst) and Glu184 (catalytic nucleophile), which play essential roles in the enzymatic reaction [9,16]. The greatest identity among sequences (54%) was obtained by comparing the sequence of HhXyl with the sequences of a β-1,4-xylanases of *Streptomyces bingchenggensis *[GenBank:ADI10429] and *S. avermitilis *[GenBank: NP_826161]. HhXyl showed amino acid sequence identities of 48%, 49%, 52% with the sequences of β-1,4-xylanases from *S. megasporus *[GenBank:ADE37527.1], *Catenulispora acidiphila *[GenBank:YP_003115119.1] and *S. scabiei *[GenBank:YP_003489277.1] respectively (Figure 1).

### Characterization of the HhXyl xylanase from coffee berry borer

**Figure 2:**
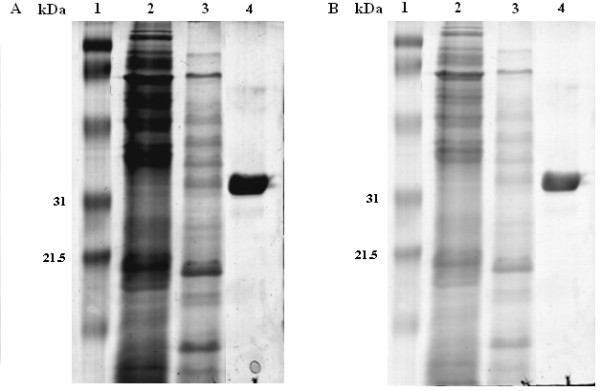
**SDS-PAGE of the purified recombinant proteins**. (A) HhXyl and (B) XIP-I from wheat. The proteins were purified by affinity chromatography using Ni-NTA resin. Lane 1: Low range weight marker (Bio-Rad), Lane 2: negative control for expression, Lane 3: Unpurified samples, Lane 4: 5 μl of the concentrated pure HhXyl (A) and XIP-I (B) proteins. The proteins were visualized with Coomassie brilliant blue R250.

The coding sequences of *HhXyl *and the xylanase inhibitor protein *XIP-I *from *T. aestivum *were cloned, without their respective signal peptides, into the pPICZαA vector, and the resulting plasmids were used to transform the GS115 strain of *P. pastoris*. Postiviely transformed colonies were confirmed by PCR and evaluated for their recombinant protein expression levels on a small scale to ultimately select those with higher yields after 96 hours of induction with methanol. The purified recombinant proteins were homogenous, as visualized on SDS-PAGE gels with single bands of the expected weights: 35.5 kDa for HhXyl, corresponding to 33 kDa of the recombinant enzyme and 2.5 kDa of a six-histidine tail (Figure 2a), and 33 kDa for XIP-I, corresponding to 29.5 kDa of recombinant XIP-I and 2.5 kDa of a six-histidine tail (Figure 2b).

**Figure 3:**
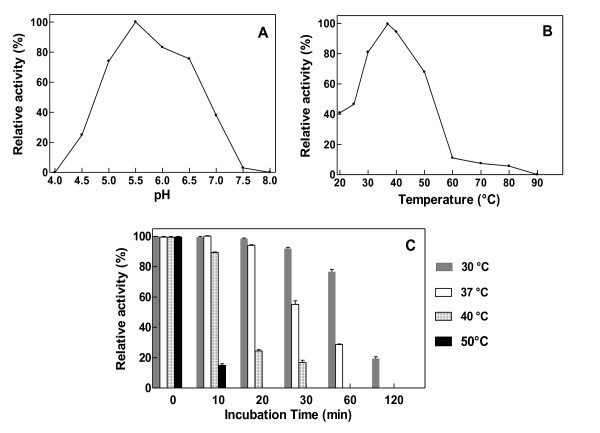
**Characterization of recombinant HhXyl**. (A) The effect of pH on the activity of HhXyL. (B) The effect of temperature on the activity of HhXyl. (C) The thermostability of HhXyl determined at different temperatures. The enzyme was incubated in the absence of of substrate for 10, 20, 30, 60 and 120 min before measuring its activity. Xylanase activity prior to pre-incubation was considered 100%.

Extracts from the digestive tracts of the borer showed hydrolytic activity on xylan polysaccharides isolated from wheat (data not shown). When the recombinant HhXyl enzyme was incubated with the xylan in solutions with a pH range between 3.0 and 8.0, maximum hydrolytic activity was observed at pH 5.5. Activity values greater than 80% of the maximal value were only detected at a pH between 5.8 and 6.0 (Figure 3a). This pH range is similar to that described for other reported xylanases [17]. Rapid hydrolysis was observed at 37°C (Figure 3b) but decreased with increasing temperature, such that at 60°C, activity was only 10% of the optimum level, and was 46% at room temperature (25°C). These conditions are similar to those of the physiological environment in which the enzyme presumably acts: approximately 37°C and acid pH in the insect's digestive system. At its optimal temperature, HhXyl maintained 58% activity after a 30 min incubation. At higher temperatures, the activity of the enzyme decreased considerably after 10 min (Figure 3c). These parameters differed from those reported for other xylanases of fungal and bacterial origin, which are active and stable at temperatures between 50°C and 70°C, and thus have the potential to be used on an industrial scale [18].

**Figure 4:**
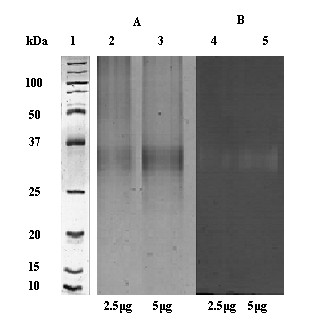
**Native-PAGE and zymogram of pure HhXyl**. Samples of pure protein were loaded on two native gels (12%). (A) In the absence of xylan, the gel was stained with Coomassie brilliant blue R250 to visualize the band corresponding to xylanase (35.5 kDa). (B) In the presence of 2% xylan, the gel was stained with Congo red to verify the xylanase activity. The amounts of protein loaded were 2.5 μg (Lanes 2 and 4) and 5 μg (Lanes 3 and 5) are indicated on the figure. The molecular weight is indicated in kilodaltons (Lane 1).

In addition, a zymogram analysis revealed that 2.5 and 5 μg of pure HhXyl enzyme produces two clear bands, with a molecular weight of approximately 35 kDa, which corresponds to the observed HhXyl weight as determined by SDS-PAGE analysis. Congo red detection indicated that the enzyme has xylanase activity against xylan isolated from wheat (Figure 4).

### Effect of XIP-I on HhXyl

**Figure 5:**
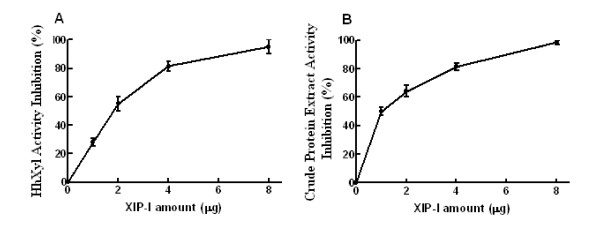
**Assay for inhibition of xylanase activity**. (A) Inhibition of HhXyl activity by different amounts of pure recombinant XIP-I. The HhXyl activity was quantified by the DNS method and xylan from oat spelt was used as a substrate. (B) Inhibition of the xylanase activity present in crude protein extracts from the digestive tracts of the borers with different amounts of pure XIP-I. The activity was quantified by the DNS method.

**Figure 6:**
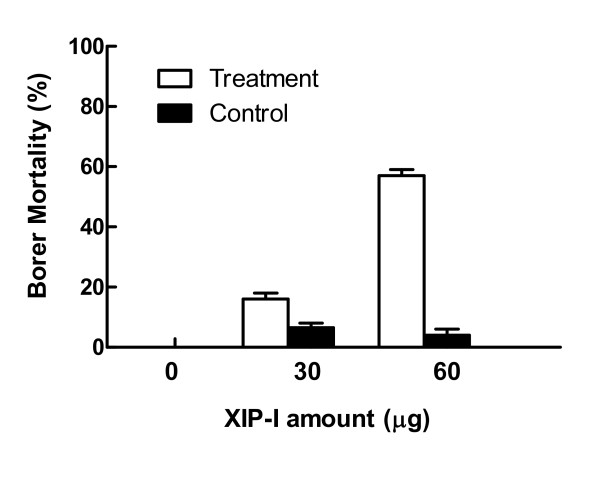
**Biological effect of XIP-I in artificial diets**. The corrected mortality rate of borer individuals due to their ingesting a Cenibroca diet supplementd with 30 and 60 μg of XIP-I for 12 days compared to control insects.

The recombinant XIP-I was tested for potential inhibitory action on HhXyl. Varying amounts of purified recombinant XIP-I inhibited xylanase activity, and while the lowest amount evaluated (2 μg) reduced activity by 50%, 8 μg resulted in 100% inhibition. A similar inhibition occurred when the recombinant protein XIP-I was incubated with borer digestive tract extracts (Figure 5). The use of XIP-I therefore represents a potential candidate for generating resistance against the coffee berry borer. In biological assays using artificial diets including 30 or 60 μg per well of XIP-I, 17% and 57% borer mortality rates, respectively, were observed after 12 days of ingestion (Figure 6). This mortality effect on *H. hampei *individuals on artificial diets supplemented with XIP-I was likely caused by reduced hydrolysis of the primary cell wall xylan polysaccharides from coffee beans that were used as a source of nutrients. In *in vitro *studies, hydrolysis of insoluble fractions of coffee beans employing recombinant β-mannanase and β-xylanase enzymes from *H. hampei*, showed a synergistic effect since the reduction of these fractions is more efficient when both enzymes are present, than when is used alone (data not shown). The fact that xylanase promotes the reduction of these fractions when added mannanase suggests that the xylanase has a role in seed polymer degradation. Inhibiting the xylanase enzyme through the addition of XIP-I in the midgut of the insects could then lead to a deterioration of the catalytic ability of mannanase, which is the enzyme responsible for degrading galactomannans, the main polysaccharide in the coffee bean.

Vasconcelos et al (2011), reported the role of a xylanase inhibitor in the germination of spores of *Phakopsora pachyrhizi*. The effect was observed in bioassays in the absence of xylan in the reaction. This suggests that such inhibitors can have important biological effects in different processes in which other glycosyl hydrolases are involved, such as cellulases and mannanases [19]. Further analysis is required to elucidate the mechanism of action.

Another possible interpretation of these data is that XIP-I may inhibit glycosyl hydrolases other than xylanases and have consequent effects on the insect. Two plant proteins named XAIP and XAIP-II, with significant sequence identity and considerable structural similarity to XIP-I, have recently been shown to bind specifically to two structurally very different hydrolases from different GH families (GH11 xylanase and GH13 α-amylase), resulting in inhibition of enzymatic activities [20,21].

XIP-I inhibits xylanases of the GH10 and GH11 families of fungal, but not bacterial origin in a reversible and competitive way. The exact nature of XIP-I biological activity is still unknown, although it is speculated to play an important role in plant defense against pathogens, and particularly those of fungal origin, because it lacks endogenous inhibitory activity against wheat xylanases [9,10,22,23]. Microbial GH enzymes are important for plant tissue invasion and degradation and so inhibitors of these enzymes are thought to play important roles in defense. It has been demonstrated that this type of inhibitor can be induced by pathogens as XIP-I is expressed strongly after inoculation with the fungus *Erysiphe graminis *and, in contrast to other inhibitors, XIP-I expression varies with the type of pathogen and the infected tissue [23].

## Conclusions

In this study a functional xylanase gene (*HhXyl*) from the digestive tracts of the coffee berry borer was cloned and expressed as a recombinant protein in a *P. pastoris *system. The enzyme has a predicted molecular mass of 34.92 kDa and contains a putative domain conserved in GH10 proteins. The enzyme showed optimal activity in similar conditions to those of the physiological medium in the borer's digestive system and was able to effectively depolymerize xylan. The xylanase inhibitor, XIP-I, from wheat was shown to have inhibitory activity against both the recombinant HhXyl enzyme and crude protein extracts from borer digestive tracts. In addition, biological assays using XIP-I supplements to artificial diets induced mortality in borer individuals. Since xylan is not a quantitatively major component of coffee beans, two possible underlying mechanisms are suggested: i) XIP-I inhibition of xylanases may influence the action of other glycosyl hydrolases in the process of digesting the major coffee bean polysaccharides that constitute the diet of the insect. ii) XIP-I may inhibit other non-xylanase proteins that are important for insect metabolism, these results indicate that use of xylanase inhibitors may provide a strategy to reducing losses caused by the coffee berry borer.

## Methods

### Isolation and preservation of coffee berry borer digestive tracts

*H. hampei *larvae were grown in dry parchment coffee with a grain humidity of 45%, at an average temperature of 27°C and 75% relative humidity in the dark. Prior to extracting the digestive tracts, larvae were incubated for 10 min at 4°C and then placed in a drop of phosphate-buffered saline (PBS), pH 7.2. For proteomic analysis, the tracts were stored in chilled PBS (pH 7.2) containing 5 mM PMSF (phenylmethylsulfonyl fluoride). Tissues used for mRNA isolation were preserved in 0.1% RNALater™ (Qiagen, Valencia, CA, USA) at -80°C.

### Complementary DNA (cDNA) library

Total RNA was extracted from 100 mg of *H. hampei *larvae digestive tracts with the RNeasy™ kit (Qiagen, Valencia, CA, USA). mRNA was isolated using an Oligotex mRNA Mini Kit (Qiagen, Valencia, CA, USA), following the manufacturer's instructions. The directional cDNA library of the borer was created with the Creator™ SMART™ Library Construction Kit (Clontech, Mountain View, CA, USA). BLAST search of the GenBank database was performed using the processed cDNA sequences, which were compared to all available ESTs and genes (http://www.ncbi.nlm.nih.gov/blast). A BLASTX results with a bit scores greater than 80 and an E-values of less than 10^-4 ^was regarded as a significant match.

### Amplification and cloning of the coffee berry borer xylanase gene and XIP-I

The partial sequence of the borer xylanase gene (*HhXyl*) was determined by amplifying genomic DNA with primers designed based on the sequences of putative xylanase genes identified in the cDNA library. The primers used for genome walking are listed in Table 1. The complete sequence of the gene and the sequences of the regions adjacent to the 5' and 3' ends were obtained using a repeated genome walking technique and nested PCR protocols from the BD GenomeWalker™ Universal kit (Clontech, Mountain View, CA, USA). The fragments produced from the genome walk were cloned into the pGEM^® ^T-easy vector (Promega, Madison, WI, USA). The sequence was analyzed with CodonCode Aligner software (V1.6.3), and homology was analyzed using BLASTX [24]. The *XIP-I *gene was amplified using genomic DNA from Soisson variety of wheat leaf tissue (*Triticum aestivum*), which had germinated for one week. The primers for amplification were as follows: XIP-I_Fw 5'-ATGGCGCCGCTCGCAGCCCG-3' and XIP-I_Rv 5'-GGCGTAGTACTTGATCAAGCTG-3', which were designed based on the registered gene sequence found in the Gemini database (http://www.ifr.ac.uk/Gemini/default.html). PCR was performed using the Advantage GC2 PCR system (Clontech, Mountain View, CA, USA) under the following conditions: 94°C for 3 min; 35 cycles of 94°C for 30 sec and 68°C for 3 min; and a final extension at 68°C for 10 min. The 915-bp product was cloned into the pGEM^® ^T-easy vector (Promega, Madison, WI, USA).

**Table 1:** Primers used in genome walking PCR

Primers	Sequence (5'-3')
GSP1_XIL_GW1_3	CCAATTCATGGAGTCGGTTTCCAATCTCAT
GSP1_XIL_GW1_5	TTCGTTGAAGATTTCGTTGACTACATCCCA
GSP2_XIL_GW1_3	GTTGATGTGGCCATTACTGAATTGGACATC
GSP2_XIL_GW1_5	GGCGTCCCATTTCATTTCATTTTCTGGTGT
GSP1_XIL_GW2_3	GATTTAGCAATTCCCGAATGATGTTCACAC
GSP1_XIL_GW2_5	CCAAGTACTTTGGCCCAGTAAACACGAAAA
GSP2_XIL_GW2_3	CAAACAGTTACACGCCGGGGTAACTCCCAT
GSP2_XIL_GW2_5	CACGGAAGCGTTTTCGAGATATGAATG
GSP1_XIL_GW3_3	CGGAATGCGATAGAAAGGGATGGTTAAC
GSP1_XIL_GW3_5	TCAAGTGTTCCCTATCAGGAAATTGAC
GSP2_XIL_GW3_3	GTCGTCATTTATAGTCTTCCCTGAAGTTG
GSP2_XIL_GW3_5	CTAGTTCGTCAACTGCACAATGCCTGT

### Heterologous expression of the *P. pastoris *system

The coding sequences for the endo-1,4-β-xylanase (HhXyl) and XIP-I proteins were cloned, without their respective signal sequences, into the pPICZαA vector (EasySelect™ *Pichia *Expression Kit, Invitrogen) generating the constructs constructs pPICZαA-XIL and pPICZαA-XIP-I respectively. The *HhXyl *gene was amplified with the forward primer XILHH_FW1_PIC 5'CCGCTCGAGAAAAGACACTTCAAAGACCATGCCAA-3' and the reverse primer XILHH_RV1_PIC 5'-TGCTCTAGACCCATTAATTGCAAGGCAGCTT-3'and the *XIP I *gene was amplified with the forward primer XIP1_FW_PIC 5'- CCGCTCGAGAAAAGAATGGCGCCGCTCGCAGCCCGGAG-3'and the reverse primer XIP1_RV_PIC 5'TGCTCTAGACCGGCGTAGTACTTGATCAAGCTG-3'. Forward primers contain the *XhoI *restriction site (underlined) and reverse primers *XbaI*. The PCR conditions were 10 cycles at 95°C for 1 min, 95°C for 25 sec, 59°C for 45 sec and a final extension of 72°C for 30 sec and 30 cycles of 95°C for 25 sec, 56°C for 45 sec, 72°C of 30 sec and a final extension of 72°C for 10 min, respectively. *E. coli *One Shot Top 10 cells (Invitrogen) were transformed with the constructs pPICZαA-XIL and pPICZαA-XIP-I. Positive clones were selected in LB media containing zeocin (25 μg/ml). The constructs were verified by restriction pattern analysis and sequencing. The GS115 strain of *P. pastoris *was transformed by electroporation at 2.0 kV, 25 μF and 200 Ω using 5 μg of recombinant plasmids that were linearized with *SacI*. After three days of growth ak 30°C, the positive colonies were selected on YPDS plates (1% yeast extract, 2% peptone, 2% dextrose, 1 M sorbitol, 2% agar and 100 mg/L zeocin). Integration of the *HhXyl *and *XIP-I *genes into the *P. pastoris *genome was confirmed by PCR using the AOX1 forward (5'-GACTGGTTCCAATTGACAAGC-3') and AOX1 reverse primers (5'-GCAAATGGCATTCTGACATCC-3').

Transformed *P. pastoris *cells were inoculated into 10 ml of buffered minimal glycerol complex media BMGY (1% yeast extract, 2% peptone, 100 mM potassium phosphate buffer [pH 6.0], 1.34% yeast nitrogen base (YNB), 0.0004% of biotin and 1% (v/v) glycerol) at 30°C and were shaken 250 rpm, until an OD_600 _between 3.0 and 6.0 was achieved. To induce HhXyl and XIP-I production, *P. pastoris *cell pellets were grown in BMMY (1% yeast extract, 2% peptone, 100 mM potassium phosphate buffer [pH 6.0], 1.34% YNB, 0.0004% biotin and 0.5% methanol) using 1/5 of the original culture media (10 ml). The cultures were incubated at 30°C and with shaking at 250 rpm. Absolute methanol was added to a final concentration of 0.5% every 24 hours for 5 days. The expression of the secreted proteins was evaluated by SDS-PAGE.

### Purification of the recombinant proteins

The recombinant proteins expressed in *P. pastoris *were purified by affinity chromatography using Ni-NTA resin (Qiagen, Valencia, CA, USA). The proteins that bound to the resin due to their tag of six-histidines N-terminal tag end were eluted with imidazole (500 mM). The eluate fractions were concentrated by ultrafiltration using an Amicon ultra-0.5, 3 kDa^® ^centrifugal filter (Millipore, Bedford, MA) and the concentrated samples were analyzed by SDS-PAGE. Protein concentrations were determined using the Bradford method and bovine serum albumin was used as a standard [25].

### Enzymatic activity assay

A total of 10 μl of HhXyl (0.5 μg/μl) was added to 490 μl of 200 mM of acetate buffer, pH 5.0, containing 4% (w/v) xylan (Sigma X0627). In addition, 10 μl of the digestive tracts extracts (0.5 μg/μl) was evaluated for xylanase activity. After incubation at 37°C for 1 hour, the amount of reduced sugars released by the reactions was quantified by the 3,5-dinitrosalicylic (DNS) method. For each 100 μl of reaction mixture, 100 μl of the Bernfeld reagent was added. The mixture was heated to boiling point, was boiled for 5 min, and the absorbance was measured at 540 nm. Xylose was used as a standard [26]. One unit of xylanase activity is defined as the amount of enzyme releasing 1 μmole of xylose equivalents per minute. All measurements were performed in triplicate.

### The effects of pH and temperature on enzymatic activity

To evaluate the effect of pH on xylanase activity, 4% (w/v) xylan from oat spelts (Sigma-Aldrich, St. Louis, MO, USA) were prepared in acetate buffer (pH 3.0 to 5.0) and phosphate buffer (pH 6.0 to 8.0). Each substrate solution was incubated with 10 μl HhXyl solution (0.5 μg/μl) at 37°C for one hour. To evaluate the effect of temperature, the activity assay was performed at pH 5.5 and at temperature ranging from 20 to 90°C. The thermostability of the enzyme was determined by measuring xylanase activity at 37°C for one hour after preincubating the enzyme for 10, 20, 30, 60 or 120 min at 30°C, 37°C, 40°C or 50°C. The xylanase activity measured prior to the preincubation at the different temperatures was considered to be 100%.

### Zymogram analysis of xylanase activity

Aliquots (2.5 and 5 μg) of HhXyl enzyme were analyzed by native PAGE (12%), according to the method described by Laemmli [27]. The protein bands were visualized with Coomassie stain. For the zymogram analysis, 2% (w/v) xylan was incorporated into the gel and electrophoresis was performed at 4°C. To evaluate the xylanase activity, the gel was incubated at pH 5.0 for one hour at 37°C. The enzymatic activity was visualized by incubating the gel in Congo red dye (0.25%) for 15 min, followed by successive washes with 1 M NaCl. To intensify the color, the gel was placed in 5% acetic acid.

### The effect of XIP-I protein on xylanase enzymatic activity

Crude protein extracts from the digestive tracts of L2 larvae and recombinant enzyme HhXyl (5 μg each one) were incubated separately for 30 min at 37°C with three amounts of purified recombinant XIP-I (2, 4 and 8 μg). Subsequently, 4% xylan was added to the reactions, and the samples were incubated for one additional hour. The percentage of XIP-I induced inhibition relative to the negative control (no inhibitor) was calculated according to equation (1):

(1) $$\%\;\text{inhibition}=100-\left(\text{O}{\text{D}}_{540}\;\text{reaction}\;\text{with}\;\text{inhibitor}*100∕\text{O}{\text{D}}_{540}\;\text{reaction}\;\text{without}\;\text{inhibitor}\right)$$

### Bioassays

The biological effects of XIP-I on the coffee berry borer were evaluated by providing the insects with a Cenibroca artificial diet [28], that was enriched with the recombinant XIP-I protein. Multiwell plates were used to provide 2 ml of food that was supplemented with 30 or 60 μg of XIP-I protein or distilled water. The borer eggs were extracted from the parchment coffee beans that were infested for 10 days, and 20 eggs were placed in each well until hatching. During the first instar larval stage (L1), a drop of the XIP-I solution or distilled water (control) was applied to the preoral cavity of each larva. The diet was maintained under controlled conditions at 27°C and 75% humidity. Each evaluation was performed in duplicate. The development of the larvae was followed daily until they reached the pupal stage when the effect of the diet on their mortality was evaluated. The mortality rate was calculated and was corrected relative to the control in accordance with Abbott's formula [29].

## Competing interests

The authors declare that they have no competing interests.

## Authors' contributions

JRG: Performed the isolation and preservation of the coffee berry borer digestive tracts. BPH and CFR: Constructed the borer cDNA library, performed the identification, amplification and cloning of HhXyl and XIP-I, as well as the subcloning in a *Pichia *vector and expression. ARS: participated in the procedures of induction of expression of the recombinant proteins. CAG, BPH and JMO: purification and characterization of HhXyl. CAG and BPH: Performed the HhXy enzymatic assays, the inhibition assays, and the bioassays. BPH, CAG and JMO: drafted the manuscript. RAZ: Conceived the study and participated in its design and coordination and helped draft the manuscript.

All authors read and approved the final version of this manuscript.
